# How Can We Support Healthy Eating in Young Adults with Low Diet Quality? A Survey of Users of the ‘No Money No Time’ Healthy Eating Website

**DOI:** 10.3390/nu14245218

**Published:** 2022-12-07

**Authors:** Megan Whatnall, Lee M. Ashton, Marc T. P. Adam, Hannah McCormick, Erin D. Clarke, Fiona Lavelle, Tracy Burrows, Melinda Hutchesson, Clare E. Collins

**Affiliations:** 1 School of Health Sciences, College of Health, Medicine and Wellbeing, University of Newcastle, Callaghan, NSW 2308, Australia; 2 Food and Nutrition Research Program, Hunter Medical Research Institute, New Lambton Heights, NSW 2305, Australia; 3 School of Education, College of Human and Social Futures, University of Newcastle, Callaghan, NSW 2308, Australia; 4 Active Living Research Program, Hunter Medical Research Institute, New Lambton Heights, NSW 2305, Australia; 5 School of Information and Physical Sciences, College of Engineering, Science and Environment, University of Newcastle, Callaghan, NSW 2308, Australia; 6 Department of Nutritional Sciences, School of Life Course & Population Sciences, King’s College London, London SE1 9NH, UK

**Keywords:** diet quality, eHealth, online, young adults

## Abstract

Nutrition interventions to support young adults are needed due to low diet quality. The aims were to explore the (1) circumstances and (2) barriers regarding dietary habits of the young adult users of the No Money No Time (NMNT) healthy eating website with the lowest diet quality scores. An online cross-sectional survey was conducted from August–September 2022 with a sample of NMNT users aged 18–35 years with low diet quality (defined as Healthy Eating Quiz score 0–38/73). The survey included demographics (e.g., gender), circumstances (6-item US Food Security Survey, Cooking and Food Skills Confidence Measures), and challenges and resources used in relation to healthy eating (open-responses). Theoretical thematic analysis was used to analyse open-response questions and derive main themes. The study sample (*n* = 108; 71.3% female, median age 28; 28.7% food insecure) had a mean (standard deviation) Cooking Skills score 70.2 (17.5)/98, and median (interquartile range) Food Skills score 96.0 (83.5–107.5)/133. The main challenges regarding healthy eating were (1) time and (2) cost, and the main resources to support healthy eating were (1) online resources (e.g., websites, Google) and (2) recipes. Findings identify possible targets for future interventions to support healthy eating in this vulnerable group (e.g., supporting cooking and food skills).

## 1. Introduction

Research has identified that young adults globally have poor diet quality compared with other adults, with this trend continuing over recent decades [1,2]. Young adulthood is typically defined as age 18 to 35 years [3], and encompasses the transition from adolescence to emerging adulthood, leading up to mid-adulthood. There is a broad range of experiences and influences on dietary behaviours during this life stage, for example undertaking tertiary study, and changes in employment and income, living situation, relationships and social environments [4]. The high rates of food insecurity and mental disorders among young adults, approximately one-third each [5,6,7,8], also impact their dietary and other lifestyle behaviours. Commonly, the impact of these experiences means poorer dietary patterns and behaviours. Their food choices are predominantly driven by convenience, cost, limited cooking and food-related skills, equipment and facilities, and/or limited nutrition knowledge [9,10,11]. Low diet quality has been reported as the norm for the majority of young adults [1,2]. Diet quality is defined as the variety of foods usually consumed from food groups aligned with recommendations in national dietary guidelines [12]. The implication of poor diet quality in young adulthood is that patterns of eating established during this stage are typically continued into adulthood, and associated with a higher risk of chronic disease, including cardiovascular disease and type 2 diabetes [13,14,15]. It is therefore a high priority to intervene and support young adults to adopt positive dietary behaviours. 

To effect positive dietary behaviour change in young adults, it is critical to involve them in the process of intervention development [16]. Co-design can facilitate this by identifying barriers, preferences and potential solutions, from the perspective of the target group, in a collaborative way that meets their needs and expectations [16], and that can be incorporated into programs including eHealth programs [17]. Co-design is important at all project stages, including updates over time. The No Money No Time (NMNT) website (https://nomoneynotime.com.au/ accessed on 9 November 2022) is a web-based program targeted to young adults, which provides evidence-based nutrition information and resources (e.g., recipes and blog articles) to support healthy eating. NMNT was developed in close consultation with young adults, and specifically focuses on time and financial constraints to achieve healthy eating [18]. These were the main barriers reported by young adults in formative work [18] and consistent with the broader literature [19]. Ongoing efforts to consult with young adults, and understand their circumstances and barriers in relation to dietary behaviours, are essential to sustain usage and engagement, and promote positive behaviour change. Further consultation with young adults with poor diet quality can support this work, as nutrition interventions targeted to this population group are needed, particularly as they are a challenging group to reach and engage in health behaviour research [19,20]. Aside from time and money, studies have identified other factors influencing eating habits in young adults, including the level of interest in healthy eating, eating habits of friends and family, desire for outcomes such as attractiveness and weight loss as a result of improved eating habits, self-motivation and self-regulation [19,21]. However, these studies have been conducted in young adults broadly, rather than specifically among those with low diet quality. Engaging with young adults with low diet quality specifically would provide invaluable insights and have broader relevance. 

The aims therefore were to explore the (1) circumstances and (2) barriers regarding dietary habits of the NMNT young adult users with the lowest diet quality scores, to provide further insight into determinants that may contribute to low diet quality. 

## 2. Materials and Methods

### 2.1. Study Design and Participants

A cross-sectional survey of a sample of users of the NMNT website was conducted. Inclusion criteria were; NMNT users who had consented to future contact, residing in Australia, aged 18–35 years, and had a diet quality score of 0–38 out of a possible 73, assessed using the Healthy Eating Quiz (HEQ) diet quality index tool [22] embedded on the NMNT website. Eligible NMNT users were identified from the user database and invited to complete the survey via email. The user database and email communications are managed using ActiveCampaign email marketing software (Chicago, IL, USA), and the survey was hosted via QuestionPro survey tool (Dallas, TX, USA). The survey was open for four weeks from the 31 August 2022 to the 28 September 2022. The initial email invitation was sent on the 31 August 2022 to *n* = 9269 NMNT users, with reminder emails sent one, two and three weeks later. To incentivise participation, completers could choose to enter a prize draw to win one of 20 $100 e-gift vouchers on survey completion. Participants could only complete the survey once to prevent multiple entries by the same person. The survey took approximately 15 minutes, and included the measures outlined below. All participants gave their informed consent before completing the survey. The study was approved by the University of Newcastle Human Research Ethics Committee (H-2018-0512). Study conduct and reporting adheres to the STROBE guidelines [23] (Supplementary Materials Table S1). 

### 2.2. Measures

#### 2.2.1. Demographics and Circumstances Regarding Dietary Habits

Demographic characteristics captured in this survey included age, gender (male/female/another gender identity), postcode of residence, and living situation. Postcode of residence was used to estimate socio-economic status, by matching postcode to indexes in the Australian Bureau of Statistics, Index of Relative Socioeconomic Advantage and Disadvantage (IRSAD) [24]. IRSAD ranges from 1/most disadvantaged to 10/most advantaged, based on the economic and social conditions of individuals and households within geographical areas. 

Food-related demographics included the person/s responsible for food shopping, and cooking, in their household, the cooking equipment available in their household, whether they had a budget for food, and what was their weekly spend on food for themselves. 

The United States Department of Agriculture (USDA), Food Security Survey Modules (FSSM) 6-item short form was used to assess food security status [25]. The tool was found to have high specificity, sensitivity and minimal bias compared with the 18-item tool, when evaluated using a nationally representative sample of US households in the 1995 Current Population Survey [26]. The six questions focus on economic access to food in the last 12 months (e.g., In the last 12 months did you ever eat less than you felt you should because there wasn’t enough money for food?), with affirmative responses scored as one point and ‘no’ responses scored as zero points. The total score range is from 0–6, with high food security classified as 0–1, low food security 2–4 and very low food security 5–6, or dichotomised to food secure (0–1) and food insecure (2–6). 

Cooking Skills Confidence and Food Skills Confidence measures were from Lavelle et al. and assessed confidence with cooking and food-related skills respectively [27]. These measures have demonstrated high internal consistency, reliability and validity over time in a sample of young adults aged 18–27 years, and in adults aged 20–60 years [27]. Both measures ask respondents to rate how good they are at each skill listed on a Likert scale from 1/Very poor to 7/Very good, or to select 0/Never or rarely do it if the skill is one they don’t do. The Cooking Skills Confidence measure assesses 14-items (e.g., Peel and chop vegetables) and individual items are summed to give a total score range of 0–98. The Food Skills Confidence measure assesses 19-items (e.g., Prepare or cook a meal with limited time) and individual items are summed to give a total score range of 0–133. Higher scores are indicative of greater confidence in cooking and food skills. 

#### 2.2.2. Barriers Regarding Dietary Habits

Two open-ended questions explored respondents’ main challenges and resources or tools used to help them to eat healthily. These specifically asked; “What are the main challenges for you when it comes to healthy eating?”, and “What are the main resources or tools that you use to help you in making healthier food choices?” These questions were optional. 

### 2.3. Statistical Analysis

Stata software version 14.2 (StataCorp., College Station, TX, USA) was used for statistical analyses. Descriptive statistics are reported as number and percentage for categorical variables, and as mean and standard deviation (SD) or median and inter-quartile range (IQR) for normally distributed and non-parametric continuous data, respectively. Qualitative analysis of open-response question data followed a theoretical thematic analysis approach [28], including (1) identifying codes from the responses based on keywords and phrases, (2) grouping codes into themes, (3) reviewing themes, and (4) defining and naming the themes. Two researchers (M.W. and H.E.) reviewed the open-response question data to identify keywords and phrases/codes and group these into themes, then discussed/reviewed and defined and named the main themes. Themes are presented in the order of most to least reported. Associations between cooking skills confidence and food skills confidence with age and gender were explored using linear regression, and are reported as β-coefficient and standard error (SE). The association of food insecurity with age and gender was explored using logistic regression, and reported as odds ratio (OR) and 95% confidence interval (CI). Age was included as a categorical variable (18–24 years; 25–29 years; 30–35 years) in regression models. Participants who responded as ‘another gender identity’ were excluded due to the small number (*n* = 2). Food security status was dichotomised as food insecure (low and very low food security) and food secure (high food security) due to low numbers with low and very low food security. Bi-variate (i.e., age or gender and the dependent variable) and multivariate (i.e., age and gender and the dependent variable) models were tested. Statistical significance was set as *p* < 0.05. 

## 3. Results

### 3.1. Description of the Study Sample

A total of 137 survey responses were received, with exclusions made due to individuals not living in Australia (i.e., ineligible) (*n* = 3), not giving consent to participate (*n* = 2), and incomplete survey responses (*n* = 24). The final study sample included 108 young adults, predominantly female (71.3%), with a median (IQR) age of 28 (24–32) (Table 1). The majority of the sample (59.2%) had an IRSAD in the middle (40.7%) or lowest (18.5%) range, i.e., moderate to low socio-economic advantage. A total of 28.7% had low (15.7%) or very low (13.0%) food security. 

### 3.2. Description of Cooking and Food Related Characteristics

Approximately half of the respondents reported that they have a food budget (51.9%) (Table 2). Most commonly, respondents reported spending $91–120 (32.4%) or $61–90 (24.1%) on food per week for themselves. Most of the respondents reported that they did the grocery shopping (84.3%) and meal preparation/cooking (87.0%) in their household. The most common food preparation/cooking equipment that respondents had where they lived were a frying pan (99.1%), stove top (98.1%) and microwave (97.2%), and the least common were slow cooker (63.9%) and barbeque (55.6%). The mean (SD) cooking skills confidence score was 70.2 (17.5) out of 98, and the median (IQR) food skills confidence score was 96.0 (83.5–107.5) out of 133. 

### 3.3. Description of Qualitative Findings; Barriers Regarding Dietary Habits

**Figure 1:**
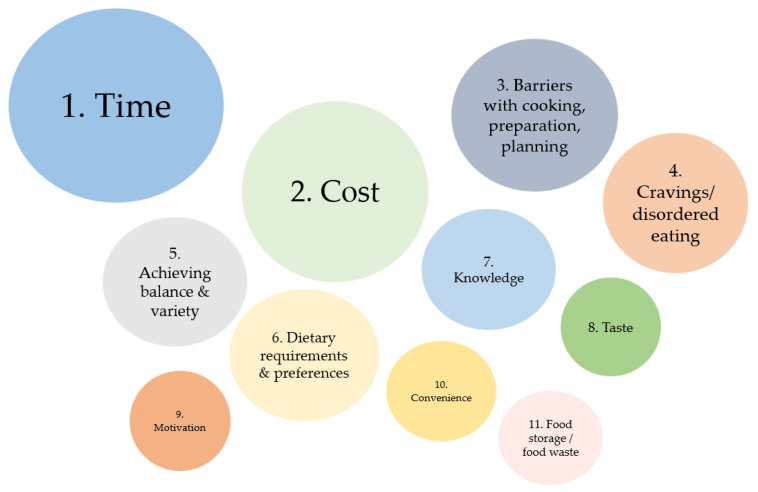
Summary of themes identified as challenges to healthy eating among young adults who use the ‘No Money No Time’ website.

Ninety-five percent (*n* = 103) of the sample responded to the question “What are the main challenges for you when it comes to healthy eating?”, with 11 themes identified from the responses. These themes are numbered in order of most to least often reported in Figure 1. Example quotes from each identified theme are provided in Table 3. 

**Figure 2:**
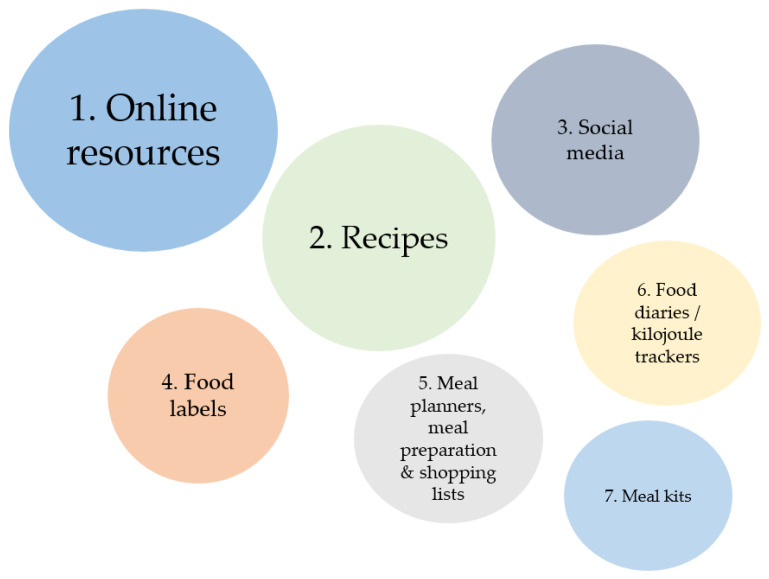
Summary of themes identified as resources or tools used to help in making healthier food choices among young adults who use the ‘No Money No Time’ website.

Ninety-three percent (*n* = 100) responded to the question “What are the main resources or tools that you use to help you in making healthier food choices?”, with seven themes identified, numbered in order of most to least often reported in Figure 2. Example quotes from each identified theme are provided in Table 3. 

### 3.4. Regression Analyses of Cooking and Foods Skills Confidence, and Food Security Status by Age and Gender

Cooking skills confidence was significantly higher in 30–35 year olds compared with 18–24 year olds in the bi-variate model (β-coefficient = 10.60, *p* = 0.011), and this remained significant in the multivariate model which also included gender (β-coefficient = 11.62, *p* = 0.006) (Table 4). Cooking skills confidence was not significantly different for 25–29 year olds compared with 18–24 year olds or by gender in bi-variate or multivariate models. Food skills confidence was not significantly different by age or gender in bi-variate models or the multivariate model. 

The odds of being food insecure were not significantly different by age or gender in bi-variate models or the multivariate model (Table 5). 

## 4. Discussion

This study explored circumstances and barriers regarding dietary habits in young adults with low diet quality. The majority of the sample were classified as moderate-low socio-economic status, most spent between $61–$120 AUD weekly on food for themselves, and almost one-third reported low or very low food security. Cooking and food skills confidence among the sample was lower than young adults in a comparable Australian study [29]. The main challenges regarding healthy eating were consistent with the broader literature, being time and cost, while the main resources used to support healthy eating were online resources and recipes. This study provides important insight into possible determinants of low diet quality in young adults, highlighting possible targets for future intervention to support healthy eating in this vulnerable group.

The majority of young adults in this study were classified as moderate-low socio-economic status, measured in terms of the economic and social conditions of the geographical areas in which they live. Further almost one third of the sample were reportedly food insecure, including 13% with very low food security. Low socio-economic status and food insecurity are both known determinants of poor dietary intake in studies of varying age and gender groups [30,31,32,33]. The findings of the current study confer with this, in a sample of exclusively young adults with low diet quality. The current study also found that most young adults were spending $61–$120 AUD per week on food for themselves, inclusive of supermarket purchases, takeaway and eating out. The average weekly spend at the supermarket for a one person household in Australia in July 2022 was $104 AUD, including predominantly food items [34]. This appears comparable, however does not account for other purchases including takeaway and eating out that would likely push this figure above that of the most common spend in the current study. Further, the average 18–29 year old Australian currently spends $154 AUD at the supermarket alone per week [34]. Overall, in terms of economic circumstances, this study suggests that low socio-economic status and food insecurity are key issues for many young adults with low diet quality. Providing financial support for individuals in this life stage is critical to improving their diet quality. As are other strategies that can mediate this relationship, such as supporting health literacy and cooking and food skills that enable individuals to maximise healthy food options with limited financial resources. 

Cooking and food skills confidence scores in the current study, mean 70/98 and median 96/133 respectively, were moderate relative to the maximum possible scores of the measurement tools used. However, these scores are lower when compared with a study of 910 Australian adults where mean cooking skills confidence among 18–29 year olds was 77, and mean food skills confidence was 106 [29]. Cooking and food skills confidence in that study were also found to increase with age, and were higher among females compared with males [29]. In the current study, a lack of significant differences were identified in cooking and food skills confidence by age and gender. However, the relatively small sample size, limited age range of 18–35 years and predominance of female participants may have contributed to this rather than there being no real differences. A key difference between the two studies is that the current study sample were specifically individuals with low diet quality, which may instead be the link to lower cooking and foods skills confidence. 

Time and cost were the two main challenges reported by young adults in the current study regarding eating healthily. This is consistent with the evidence base in this area over the last 5–10 years [19,21], and suggests that ongoing efforts are needed to address these key barriers. Several other themes that were identified in terms of challenges with healthy eating can be linked to food skills, including barriers with planning, knowledge, food storage and food waste. While other barriers related to cooking skills were also identified, such as knowing how to prepare and cook foods, and these skills are necessary to produce meals, it may be that food skills such as planning and using leftovers to prevent food waste are more important in this age group [19]. For example, where young adults may have limited food storage space when living in share houses or university residences, food skills such as planning how much to buy would be pertinent. Similarly, skills such as using leftovers to create another meal are critical to prevent food waste. This would also help to save money that is otherwise spent on wasted food, which is even more important in current times with increases in the cost of living. While not all the challenges identified relate to cooking and food skills, several of them do, and therefore lower skills and confidence in food and cooking may be a contributor to their vulnerability to low diet quality. 

Cravings for unhealthy foods and experiences of disordered eating was another theme identified in the current study as being a challenge for eating healthily. Within this theme, young adults described problems with overeating and binge eating, and a lack of self-control around unhealthy foods. Age 12–25 years is recognised as the peak onset for eating disorders [35], which crosses into the young adult life stage. While eating disorders comes under the classification of mental disorders [36], it is important to recognise the association here with dietary intake. Where most of the barriers reported by young adults in this study were practicality and resource focused, this theme of cravings and disordered eating is psychological. Evidently, the challenges surrounding achieving a healthy diet in young adulthood are multi-faceted, and the strategies required to support young adults need to reflect this. 

Some of the main resources young adults reported using to help them in making healthier food choices were online resources (such as Google, websites, apps and online articles), recipes, referring to social media, and meal planning and preparation. These identified resources share some similarities with factors that have been identified as facilitators of cooking meals at home, including the ability to plan and prepare meals ahead and having inspiration for what to cook [37]. Other resources identified in the current study, including the use of food labels, and food diaries and kilojoule trackers, are more specifically health oriented. These match with previous scoping review findings of motivations for consuming a healthy diet among young adults, including the desire for improved health, to manage weight, to boost self-esteem, and desire for attractiveness to others [19]. 

The findings from this study highlight possible targets for interventions and strategies to support healthy eating in young adults, including those with low diet quality. These can be used within the No Money No Time website, and other interventions targeting young adults. The key targets include financial support, and supporting the development of cooking and food skills and confidence. Aside from monetary support, financial support can encompass more knowledge and skill development such as teaching health literacy and cooking and food skills that enable healthy eating within financial constraints. For example, how to budget for food costs and limit food waste. Additionally, supporting young adults to develop their cooking and food skills, and in turn their confidence in these areas, may help to improve their dietary habits and diet quality. For example, instruction in basic food preparation and cooking methods, and how to prepare meals with few ingredients or limited time. Critically, the way that interventions and individual components are framed and delivered needs to align with the needs and preferences of young adults, i.e., tailored and targeted approach, in order to engage them and have the desired effect. The findings of this study highlight that online resources are the preferred choice, time is a key factor, and barriers such as the psychological aspects of eating, motivation and convenience are important considerations for this group. 

The main strength of this study is the sample exclusively being those with low diet quality, as these are a harder to reach sample. Further, the high proportion of the sample with low to moderate socio-economic status and experiencing low or very low food security add strength, as this gives important insight into the circumstances and barriers of these vulnerable groups. Additionally, validated tools were used to assess cooking and food skills and food security status, and a robust method used for qualitative analyses. Limitations to be considered include the low response rate and therefore small sample size. Although NMNT users receive regular emails through the Active Campaign software, a high proportion of emails sent may have been identified as spam or junk and not actually received. By marketing standards however, the conversion rate (i.e., number completed/number invited) of 1.2% is comparable with email marketing campaigns in the health and fitness category [38]. The small sample size and low numbers for some demographic characteristics may have contributed to the lack of statistically significant findings in regression models, and limits the utility of more in-depth statistical approaches e.g., clustering at this time [39]. Further, the data being self-report introduces potential self-report bias. 

## 5. Conclusions

Nutrition interventions to specifically support young adults with low diet quality are needed. These should be targeted and tailored, considering their unique circumstances and barriers regarding healthy eating, including food security, socio-economic status and cooking and food skills. Key targets for interventions to support healthy eating in young adults include financial support, and support related to cooking and food skills. Interventions should also use young adults’ preferred method of delivery and resources, including online resources, recipes and social media.

## Figures and Tables

**Table 1:** Demographics of young adults with low diet quality who use the ‘No Money No Time’ website (*n* = 108).

Demographic Characteristic	*n* (%) ^a^
**Gender**	
Female	77 (71.3)
Male	29 (26.9)
Another gender identity	2 (1.9)
**Age (years), median (IQR)**	28.0 (24.0–32.0)
18–24 years	29 (26.9)
25–29 years	36 (33.3)
30–35 years	43 (39.8)
**Living situation**	
Own my home	39 (36.1)
Parent/s home	29 (26.9)
Renting with others (e.g., partner, friends)	32 (29.6)
Renting on my own	6 (5.6)
University/College accommodation	1 (0.9)
Community/Departmental housing	0 (0)
Other	1 (0.9)
**Index of relative socio-economic advantage and disadvantage (decile)**	
1–3 (most disadvantaged)	20 (18.5)
4–7	44 (40.7)
8–10 (most advantaged)	44 (40.7)
**Food security status**	
High food security	77 (71.3)
Low food security	17 (15.7)
Very low food security	14 (13.0)

^a^ Values are *n* (%) unless otherwise specified. IQR, interquartile range.

**Table 2:** Cooking and food related characteristics of young adults with low diet quality who use the ‘No Money No Time’ website (*n* = 108).

Characteristic	*n* (%) ^a^
**Do you have a food budget**	
Yes	56 (51.9)
No	52 (48.1)
**Approximate food spend per week for themselves**	
$0–30	4 (3.7)
$31–60	13 (12.0)
$61–90	26 (24.1)
$91–120	35 (32.4)
$121–150	17 (15.7)
More than $150	13 (12.0)
**Who does grocery shopping in the household ^b^**	
Me	91 (84.3)
Partner	35 (32.4)
Parent/s	23 (21.3)
Roommate/s	5 (4.6)
Other	2 (1.8)
**Who does meal preparation/cooking in the household ^b^**	
Me	94 (87.0)
Partner	14 (13.0)
Parent/s	24 (22.2)
Roommate/s	7 (6.5)
Other	4 (3.6)
**Food preparation/cooking equipment available where you live ^b^**	
Frying pan	107 (99.1)
Stovetop	106 (98.1)
Microwave	105 (97.2)
Kettle	104 (96.3)
Oven	103 (95.4)
Pot	102 (94.4)
Toaster	99 (91.7)
Blender	84 (77.8)
Sandwich press	83 (76.9)
Slow cooker	69 (63.9)
Barbeque	60 (55.6)
Other	10 (9.0)
**Cookings skills score/98, mean (SD)**	70.2 (17.5)
**Food skills score/133, median (IQR)**	96.0 (83.5–107.5)

^a^ Values are *n* (%) unless otherwise specified. ^b^ Sum of percentages > 100 as respondents could select multiple options. SD, standard deviation. IQR, interquartile range.

**Table 3:** Summary of challenges and resources used in relation to healthy eating among young adults with low diet quality who use the ‘No Money No Time’ website.

Question: What Are the Main Challenges for You When It Comes to Healthy Eating? ^a^	
**Themes and quotes**	**Time;** “time and being busy to go and buy ingredients to make a healthy meal”.
	**Cost;** “Usually it’s the price point of the healthy option compared to the not so healthy option”.
	**Barriers with cooking/preparation/planning**; “Difficult to cook meals for one person and gets boring if meal prepping to eat leftovers”.
	**Cravings/disordered eating**; “the temptation of eating unhealthy”. **Achieving balance and variety**; “Variety. I have a few healthy cheap meals I know but I get bored with them”.
	**Dietary requirements and preferences**; “Dietary requirements of the household and personal preferences of other people I cook for”
	**Knowledge**; “…Knowledge—I like to reduce the amount of meat I cook, but I don’t always have inexpensive ideas on how to make veg and legumes the main food item in meals”
	**Taste**; “I enjoy cooking and food, and unfortunately making food taste delicious often includes high amounts of fats/salt”
	**Motivation;** “…motivation when feeling tired after work” **Convenience;** “Ensuring there are quick methods to get veggies into me is hard when chips etc. are so easy to put in the oven.”
	**Food storage/food waste;** “…finding recipes that taste good and have the correct balance of different foods to have in a meal (without having to worry about wasting fresh ingredients that go off)”
**Question: What are the main resources or tools that you use to help you in making healthier food choices? ^b^**	
**Themes and quotes**	**Online resources;** “…basic online articles outlining the benefits and pitfalls of particular ingredients”
	**Recipes (books and online);** “Google healthy recipes using what’s in our fridge at the time…”
	**Social media;** “…Instagram accounts that post ‘healthy swaps’”
	**Food labels;** “Read nutrition panels on food.”
	**Meal planners, meal preparation and shopping lists;** “Meal planning on the weekend and writing a shopping list”
	**Food diaries/kilojoule trackers;** “Food diary and working out how much food I should be eating daily”
	**Meal kits;** “We have started using HelloFresh”

Open-response questions were optional to complete; **^a^** *n* = 103 responses, **^b^** *n* = 100 responses. Themes are presented in order of most to least often reported.

**Table 4:** Multivariate linear regression results of cooking and food skills confidence with age and gender among young adults with low diet quality who use the ‘No Money No Time’ website (*n* = 106).

Variable	β-Coefficient	SE	*p*-Value
**Cooking skills confidence**			
Gender (Reference category = Male) ^a^			
Female	1.23	3.68	0.74
Age (Reference category = 18–24 years)			
25–29 years	2.71	4.25	0.53
30–35 years	11.62	4.12	0.01
**Food skills confidence**			
Gender (Reference category = Male) ^a^			
Female	0.81	4.59	0.86
Age (Reference category = 18–24 years)			
25–29 years	−3.06	5.29	0.56
30–35 years	5.24	5.13	0.31

β-coefficient is the increase in cooking or food skill confidence for each category of gender and age compared with the reference. ^a^ *n* = 106; respondents who reported ‘another gender identity’ were not included in the model due to the small number (*n* = 2). SE, standard error.

**Table 5:** Multivariate logistic regression results of food security status with age and gender among young adults with low diet quality who use the ‘No Money No Time’ website (*n* = 106).

Variable	Odds Ratio	95% CI	*p*-Value
**Gender (Reference category = Male) ^a^**			
Female	2.37	0.80, 7.02	0.12
**Age (Reference category = 18–24 years)**			
25–29 years	1.02	0.36, 2.89	0.97
30–35 years	0.43	0.14, 1.31	0.14

Odds ratio is the odds of being food insecure for each category of gender and age compared with the reference. ^a^ *n* = 106; respondents who reported ‘another gender identity’ were not included in the model due to the small number (*n* = 2). CI, confidence interval.

## Data Availability

Data used in this study is not publicly available due to rules and restrictions of the approving Ethics Committee.

## Supplementary Materials

The following supporting information can be downloaded at: https://www.mdpi.com/article/10.3390/nu14245218/s1, Table S1: STROBE Statement—Checklist of items that should be included in reports of cross-sectional studies.
